# Relationship between QT Interval Length and Arterial Stiffness in Systemic Lupus Erythematosus (SLE): A Cross-Sectional Case-Control Study

**DOI:** 10.1371/journal.pone.0152291

**Published:** 2016-04-11

**Authors:** Ricardo Rivera-López, Juan Jiménez-Jáimez, José Mario Sabio, Mónica Zamora-Pasadas, José Antonio Vargas-Hitos, Josefina Martínez-Bordonado, Nuria Navarrete-Navarrete, Ricardo Rivera Fernández, E. Sanchez-Cantalejo, Juan Jiménez-Alonso

**Affiliations:** 1 Cardiology Clinical Management Unit, Granada University Hospitals; Granada Institute of Biohealth Research.Granada. Spain; 2 Systemic Autoinmune Diseases Unit. Department of Internal Medicine. Granada University Hospitals; Granada Institute of Biohealth Research.Granada. Spain; 3 Intensive Care Unit. Serrania Hospital, Ronda (Málaga). Spain; 4 Andalusian School of Public Health, Granada, Spain; 5 CIBER de Epidemiología y Salud Publica (CIBERESP), Madrid, Spain; 6 Instituto de Investigación Biosanitaria de Granada (Granada. ibs), Granada, Spain; Renal Division, Peking University First Hospital, CHINA

## Abstract

**Introduction and Objectives:**

The QT interval on the electrocardiogram has been shown to be longer in patients with systemic lupus erythematosus (SLE) compared to that of the general population. The clinical significance of this finding is unknown. The aim of this study was to assess the relationship between QT interval and subclinical atherosclerosis, measured by carotid-femoral pulse-wave velocity.

**Material and Methods:**

93 patients with SLE and 109 healthy women with similar basal characteristics were studied. All patients underwent a 12- lead electrocardiogram, and corrected QT interval (QTc) was measured using the Bazett’s formula. The presence of atherosclerosis was evaluated by carotid-femoral pulse-wave velocity.

**Results:**

Clinical basal characteristics were similar in both groups. QTc interval was 415±21.4 milliseconds in all patients, and 407±19.1 milliseconds in the control group (p = 0.007). There was a positive correlation between QTc interval and carotid-femoral pulse-wave velocity (r = 0.235; p = 0.02) in patients with SLE. This association was independent of hypertension and age in a multivariate analysis.

**Conclusion:**

QTc interval measured by electrocardiogram is prolonged in SLE patients; it is related to subclinical atherosclerosis, measured by carotid-femoral pulse-wave velocity. This measure may help stratify risk in routine clinical practice and select the patients that might benefit from a more aggressive therapy in the prevention of cardiovascular events.

## Introduction

The prevalence of atherosclerosis is higher in patients with systemic lupus erythematosus (SLE) than in the general population, becoming today a leading cause of morbidity and mortality in these patients [1, 2]. Identifying SLE patients with high cardiovascular risk is key to tackling this problem. A prolonged QT interval on the electrocardiogram (ECG) is an easily measurable, reproducible parameter that has been linked with early-onset atherosclerosis in the general population and some subpopulations with high cardiovascular risk [3–6]. SLE patients also appear to have a more prolonged QT interval, although clinical relevance of this has not been studied [7]. Carotid-femoral pulse wave velocity (PWV) is a fairly precise indirect measure of subclinical atherosclerosis, and several studies have reported a strong correlation with coronary angiography and cardiovascular mortality during patients’ follow-up [8, 9]. Carotid-femoral PWV has also been used to assess the presence of atherosclerosis in patients with SLE and other rheumatic disorders [10–12]. However, the relationship between prolonged QT interval and clinical or subclinical atherosclerosis in SLE patients has not been demonstrated.

Our aim was to correlate the prolonged QT interval on the ECG with the presence of subclinical atherosclerosis measured using a non-invasive technique, such as PWV.

## Materials and Methods

### Study population

All women with a definitive diagnosis of SLE, fulfilling at least four of the American College of Rheumatology criteria [13] and at least one year of clinical history treated at our systemic disease Unit were enrolled in the study. In addition, a control group matched for sex, age, and education level was recruited. A smaller proportion of controls were recruited from the investigators’ acquaintances. Patients with evidence of significant cardiopathy were excluded (history/evidence of acute myocardial infarction, symptoms of heart failure, murmur suggestive of significant valvular heart disease, long-term pericarditis). Patients with an exogenous correctable cause of prolonged QTc interval (e.g. medication, electrolyte disturbances) and patients in atrial fibrillation or with left bundle branch block were also excluded. The study was approved by the Local Ethics Committee. All subjects who agreed to participate in the study signed the informed consent.

### Variables and Study Design

We conducted a cross-sectional case-control study, in which we compared the QTc interval on the ECG with the carotid-femoral PWV obtained at the same time point.

**Fig 1:**
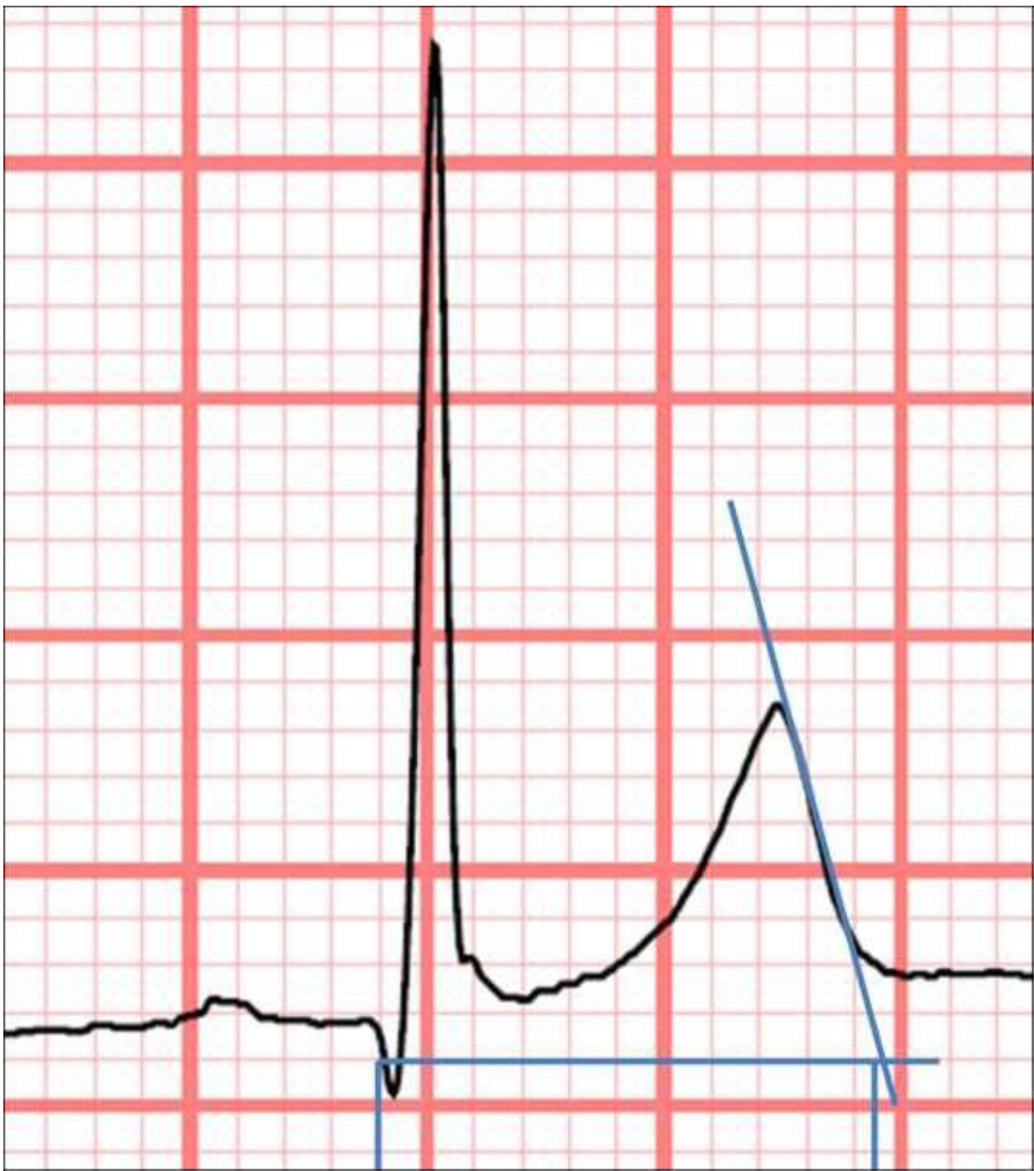
QT interval measurement according to the tangent method.

The ECGs were taken using the same electrocardiograph in patients and control subjects at a paper speed of 25 mm/s and amplitude of 10 mm/mV. We preferably used lead II, and V5 if needed, to measure the QT interval. Fig 1 shows the way QT interval was measured, starting at the beginning of the Q wave and using the tangent method described by Postema et al [14]. Two observers calculated the average length of three consecutive beats with a similar previous RR interval, correcting it using the Bazett’s formula to obtain the QTc interval [15–17]. Finally, the observers’ concordance was assessed with the Bland-Altman test and the intraclass correlation coefficient test. Other ECG parameters were also measured (see Table 1).

**Table 1:** Baseline clinical and ECG characteristics of lupus patients and control subjects.

	Lupus N = 93	Control group N = 109	P value
Age	37.99+11.3	37.81 ± 11.1	0.90
BMI (Kg/m^2^)	24.68±4.5	24.12±6.3	0.47
SBP (mm Hg)	119.3±13.2	114.84±12.0	0.13
DBP (mm Hg)	75.16±9.8	72.25±9.7	0.36
PWV (m/s)	7.66+1.1	7.12+1.0	0.001
Disease Duration (years)	11.59±7.4		
SLEDAI score	2.37±2.3		
Smoker*	23 (24.7%)	35 (32.1%)	0.15
Menopause*	26 (27.9%)	20 (19.2%)	0.09
Sedentary*	49 (46.2%)	58 (53.2%)	0.52
Dyslipidemia*	44 (47.3%)	44 (41.9%)	0.39
Chronic Kidney Disease*	12 (12.9%)	0 (0%)	0.0001
Regular prednisone use*	54 (58.0%)		
Hydroxychloroquine use*	84 (90.3%)		
Diabetes mellitus*	1 (1%)	0 (0%)	0.46
Non-specific repolarization abnormalities*	13 (13.9%)	13 (12.3%)	0.41
First-degree AV block*	9 (9.6%)	4 (3.6%)	0.07
RBBB *	4 (3.8%)	2 (1.8%)	0.41
Metabolic syndrome*	16 (17.2%)	4 (3.6%)	0.02

BMI: body mass index; SBP: systolic blood pressure; DBP: diastolic blood pressure; PWV: pulse wave velocity; SLEDAI: Systemic Lupus Erythematosus Disease Activity Index; AV: atrioventricular; RBBB: right bundle branch block.

* X^2^was used for qualitative variables

Arterial stiffness was assessed by measuring carotid-femoral PWV using an automatic device (CompliorAnalyse®, Alam Medical, Vincennes, France), operated by a single observer blinded to the patient's information. A tonometry system that automatically detects the pulse waveforms of the right common carotid and right femoral arteries was used in patients in the supine position [18]. Two measurements were performed and the mean value was taken. If the difference between the two measurements was more than 0.5 m/s, a third measurement was performed and the median value was taken according to expert consensus recommendations [19].

Other data were also collected from the patient histories: age, sex, disease duration, height, atherosclerosis risk factors (smoking, hypertension, diabetes, dyslipidemia (LDL, HDL), renal failure or nephrotic syndrome, triglycerides, hemoglobin, anti-DNA antibodies, and serum complement). The presence of metabolic syndrome was determined using the National Cholesterol Education Program Adult Treatment Panel III criteria [20]. Disease activity and accrual of organ damage were measured using the Safety of Estrogens in Lupus Erythematosus National Assessment version of the Systemic Lupus Erythematosus Disease Activity Index (SLEDAI) [21] and the Systemic Lupus International Collaborating Clinics/American College of Rheumatology Damage Index (SDI) [22], respectively.

### Statistical analysis

The continuous variables were expressed as mean±standard deviation. The qualitative variables were expressed as absolute and relative frequencies. The Student's t test was used to compare the means of the SLE patients and the controls. The correlation between the quantitative variables was analyzed using the Pearson correlation test. The Bland-Altman plot and the intraclass correlation coefficient were used to analyze the agreement between the QTc interval measurements of the two observers. Multivariate analysis was performed with multiple linear regression for continuous dependent variables and with multiple logistic regression for qualitative dependent variables with two categories. A two-sided p<0.05 was considered statistically significant. All analyses were done using SPSS 21.0 (Inc., Chicago, USA).

## Results

Ninety three women with SLE and 109 control subjects were studied. Table 1 shows the baseline characteristics of the two groups. No statistically significant differences were found in terms of ECG abnormalities with the exception of the QTc interval.

**Fig 2:**
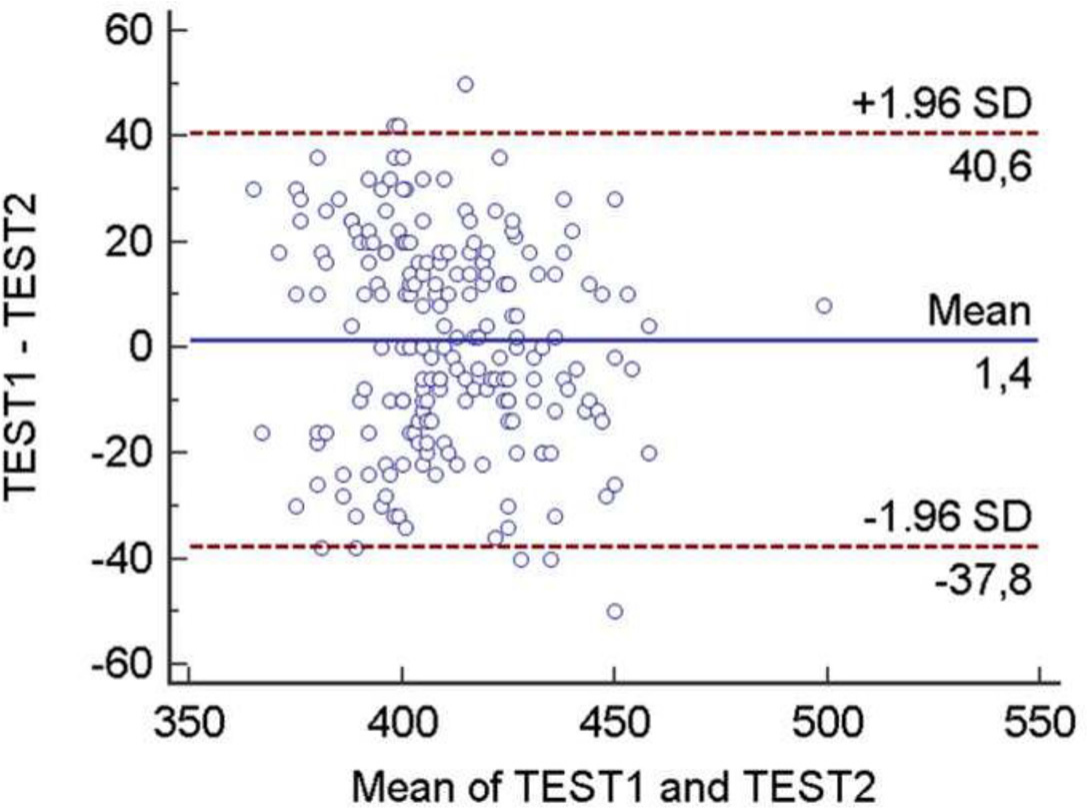
Bland Altman test showing excellent concordance between both observers.

**Fig 3:**
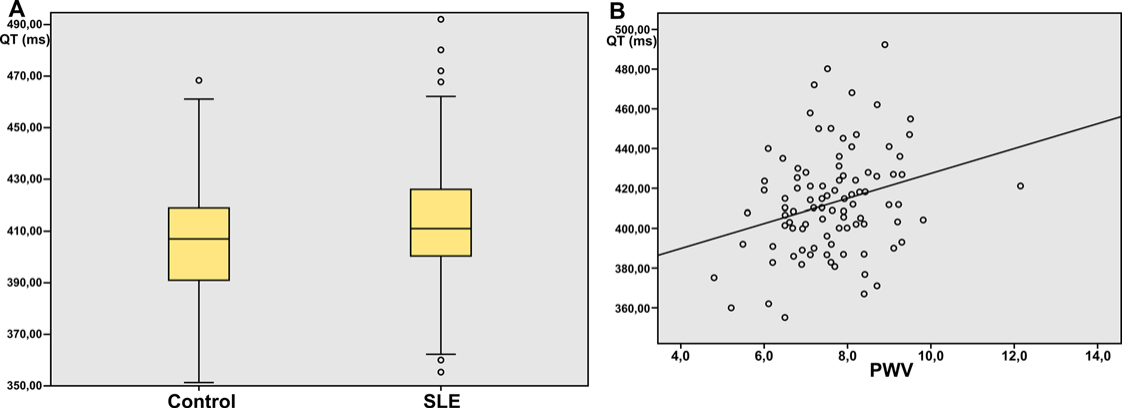
A. Comparison of QTc interval in SLE patients and control subjects. B: Positive correlation between QTc interval and wave pulse velocity.

Concordance between QTc measurements from both observers was excellent, according to the Bland-Altman plot test (Fig 2): the agreement was very high with an intraclass correlation coefficient of 0.764 (0.689–0.821). The QTc interval was significantly prolonged in SLE patients with respect to the control subjects (Fig 3A, 415±21.4 ms vs. 407±19.1 ms, p = 0.007). Additionally, statistically significant differences were found between SLE patients and controls in subclinical atherosclerosis assessed with carotid-femoral PWV (7.66±1.1 m/s vs 7.12±1.1 m/s, p = 0.001, Table 1). A positive correlation between PWV and QTc (r = 0.263, p<0.001) was found in the 202 subjects. Similarly, in the 93 SLE patients, there was a statistically significant link between QTc interval length and the presence of subclinical atherosclerosis measured using carotid-femoral PWV. The scatterplot in Fig 3B shows this positive correlation (r = 0.235, p = 0.02).

A bivariate analysis was performed in SLE patients using variables that might be related to carotid-femoral PWV (Table 2). Only those variables with a significant relationship with carotid-femoral PWV were then analyzed in the multivariate analysis with multiple linear regression. Furthermore, a comparison of the carotid-femoral PWV between patients with positive and negative Ro/SSA autoantibodies was done, and no statistically significant relationship was found (P = 0.34).

**Table 2:** Relationship between pulse wave velocity (PWV) and other variables in 93 patients with SLE.

	PWV	P value
Age< = 45 Age>45	7.34+/-0.97 8.37+/-1.17	<0.001
Smoker Non smoker	7.97+/-1.20 7.56+/-1.10	0.134
Menopause No menopause	8.39+/-1.14 7.38+/-1.01	<0.001
Sedentary No sedentary	7.56+/-1.23 7.56+/-1.02	0.406
Dyslipidemia No Dyslipidemia	7.84+/-1.28 7.50+/-0.98	0.153
Chronic Kidney Disease No Chronic Kidney Disease	8.34+/-1.10 7.55+/-1.11	0.017
Prednisone use No prednisone use	7.50+/-1.22 7.89+/-0.97	0.105
Hydroxychloroquine use No Hydroxychloroquine use	7.60+/-1.15 8.21+/-0.83	0.074
Metabolic syndrome No Metabolic syndrome	8.42+/-1.49 7.50+/-0.99	0.017
BMI*	0.293	0.004
SBP (mm Hg)*	0.334	0.001
SLEDAI*	-0.192	0.065
QTc*	0.236	0.023

BMI: body mass index; SBP: systolic blood pressure; DBP: diastolic blood pressure; PWV: pulse wave velocity; SLEDAI: Systemic Lupus Erythematosus Disease Activity Index.

* Pearson coefficient correlation was calculated between continuous variables

In the 93 SLE patients, the multivariate analysis with multiple linear regression (Table 3) showed that QTc interval prolongation was an independent predictor of the presence of subclinical atherosclerosis measured with PWV. Thus, according to the multiple linear regression, the carotid-femoral PWV value was linked to the QTc interval (B: 0.01±0.005), age, and systolic blood pressure (SBP), but not with metabolic syndrome nor with chronic kidney disease. The model's coefficient of determination (R^2^) was 0.28.

**Table 3:** Multivariate analysis with multiple linear regression in lupus patients. Dependent variable: PWV.

	B coefficient	Partial coefficient correlation	Standard error B	P
Systolic blood pressure	0.022	0.290	0.008	0.005
Age>45*	0.89	0.391	0.22	0.000
QTc interval	0.01	0.210	0.005	0.046

* Age≤45 = 0, age>45 = 1

Another multivariate analysis with logistic regression was done with the whole SLE and control sample. This statistical study was aimed to assess whether the more prolonged QTc interval observed in SLE patients might be due to a higher proportion of subclinical atherosclerosis. The logistic regression analysis (Table 4, Model 1) showed a statistically significant relationship between having SLE or being a control (dependent variable) and the QTc interval (OR = 1.02 (1.005–1.034), p = 0.008). Subsequently, the carotid-femoral PWV variable was added to the model (Table 4, Model 2), and the relationship between the dependent variable (lupus/control) and the QTc interval measurement ceased to be statistically significant (OR = 1.014 (0.999–1.030), p = 0.057); the relationship with carotid-femoral PWV was statistically significant (OR: 1.498 (1.13–1.98), p = 0.005). Finally, we checked whether adding other variables to the model such as SBP and metabolic syndrome could modify it (Table 4, Model 3). Carotid-femoral PWV remained statistically significant (OR: 1.387(1.035–1.857), p = 0.028); also, a statistically significant relationship was found in the metabolic syndrome (OR: 3.8 (1.18–12.6), p = 0.026). SBP (p = 0.374) and QTc interval were not included in the model due to the lack of statistical significance.

**Table 4:** Multivariate Analysis (Logistic Regression): Variables related to Systemic lupus erythematosus (SLE) or being a control for different models studied.

Model	Variable	OR	P
Model 1 QTc	QTc	1.02 (1.005–1.0034)	0.008
Model 2 QTc- VOP	QTC	1.014 (0.999–1.030)	0.057
Model 2 QTc- VOP	VOP	1.498 (1.131–1.984)	0.005
Model 3 QTc-VOP-Met syndrome	QTc	1.015 (0.999.-1.030)	0.058
Model 3 QTc-VOP-Met syndrome	VOP	1.387 (1.035–1.857)	0.028
Model 3 QTc-VOP-Met syndrome	Metabolic Syndrome	3.849 (1.176–12.596)	0.026

## Discussion

In our study, SLE patients had a longer QTc interval than the control subjects; there was a correlation between a longer QTc interval and the presence of subclinical atherosclerosis. This was measured using carotid-femoral PWV while controlling for age and hypertension, which are the two variables that have most influenced carotid-femoral PWV. As far as we know, this is the first time this link in SLE patients has been described in the literature. These preliminary results suggest that a simple, inexpensive and reproducible measurement of the QTc interval, combined with other clinical, biochemical and imaging data, could help to identify SLE patients with a higher degree of subclinical atherosclerosis. Further studies are required to confirm our findings in order to establish how useful this technique would be in routine clinical practice.

In the general population, a prolonged QTc interval has been found to be a predictor of cardiovascular events such as arrhythmias [23]. However, this link has not been studied in depth in SLE patients. In a case-control study, Cardoso et al. found that SLE patients had a longer QTc interval than control subjects, as per our findings. Furthermore, an independent link was found between this longer QTc interval in SLE patients and the presence of electrocardiographic left ventricular hypertrophy, non-specific ST-T-wave abnormalities, and left atrial enlargement [7]. Previous studies have reported a 15% prevalence of QTc interval prolongation in patients with SLE [24]. However, authors did not investigate the link with subclinical atherosclerosis, as we did. In patients with rheumatoid arthritis, a 50-msec increase in QTc interval was associated with a doubling of the hazard for all-cause mortality [12]. In addition, a link has been recently found between inflammatory cytokines and QTc interval in patients with rheumatoid arthritis, suggesting that a lower inflammatory burden may protect against QTc prolongation in these patients [25]. Other studies have detected a significant relationship between carotid-femoral PWV and the presence of anti Ro autoantibodies. However, this finding has not been confirmed in other studies [26]; in our case, no a significant association between the two variables was found.

It is difficult to determine the clinical relevance of our findings. After adjusting for age and hypertension, being the most important carotid-femoral PWV-related factors [8], the B coefficient of the link between QTc interval and PWV was 0.01. In other words, every 100 msec increase in the QTc interval was associated with a mean 1 m/s associated increase in carotid-femoral PWV. So a 100 msec difference in the QTc interval would be similar, in terms of carotid-femoral PWV, to a 45 mmHg difference in SBP, all other parameters in the multivariate analysis being equal, given that the B coefficient for SBP was 0.022. In the logistic regression analysis between QTc and SLE, the OR decreased when carotid-femoral PWV was introduced, and the link ceased to be statistically significant. Table 4 shows three logistic regression models: the observed changes between the first and the second model regarding QTc interval (OR decrease, changes in P value and lack of statistical significance with the dependent variable) support the hypothesis that the differences observed in QTc interval length between the SLE patients and the control group might be due to a higher degree of subclinical atherosclerosis, assessed with carotid-femoral PWV. However, we cannot assure causality between both variables due to the cross-sectional design of the study. These multivariate results did not change in spite of adding other variables that might be potential confounders to the model.

Several limitations should be considered. Our cohort was relatively small. Even so, the statistical power was enough to attain statistical significance in the correlation between QTc interval and carotid-femoral PWV after adjusting for confounding variables. Also, subclinical atherosclerosis was detected using carotid-femoral PWV, which is an indirect measurement. However, carotid-femoral PWV has been largely validated as a reliable way to assess subclinical atherosclerosis, not only in the general population, but specifically in patients with SLE and other rheumatic disorders [8–11]. Since this was a cross-sectional study, no causal relationship between the two factors can be established. Similarly, since there is no prospective follow-up, it is unknown whether having a more prolonged QT interval could lead to a higher rate of cardiovascular events in the long term. Therefore, further prospective studies testing this possibility are required.

## Conclusion

The QTc interval measured on the ECG is prolonged in SLE patients, with a statistically significant correlation with subclinical atherosclerosis measured using carotid-femoral PWV. This measure could help stratify risk in routine clinical practice and select patients who might benefit from more aggressive treatment to prevent cardiovascular events; however, these findings should be confirmed in prospective studies.
